# Functional and Survival Outcomes of Partial Versus Radical Nephrectomy in Renal Cell Carcinoma Patients With Pre-Existing Chronic Kidney Disease: A Real-World Study

**DOI:** 10.14740/wjon2728

**Published:** 2026-03-05

**Authors:** Luan Kinh Thai, Viet Quang Luu, Sam Minh Thai

**Affiliations:** a University of Medicine and Pharmacy at Ho Chi Minh City, Cho Lon Ward, Ho Chi Minh City, Vietnam; b Cho Ray Hospital, Cho Lon Ward, Ho Chi Minh City, Vietnam

**Keywords:** Renal cell carcinoma, Partial nephrectomy, Radical nephrectomy, Chronic kidney disease, eGFR, Overall survival

## Abstract

**Background:**

Managing renal cell carcinoma (RCC) in patients with pre-existing chronic kidney disease (CKD) or a solitary kidney requires balancing oncologic control with maximal renal functional preservation. This study analyzes long-term renal function, survival, and complications between partial nephrectomy (PN) and radical nephrectomy (RN) in this high-risk Vietnamese cohort.

**Methods:**

We retrospectively reviewed 90 patients with RCC and reduced pre-operative estimated glomerular filtration rate (eGFR, < 60 mL/min/1.73 m^2^) or a solitary kidney who underwent surgery at Cho Ray Hospital between 2019 and 2024. Outcomes included changes in eGFR, CKD stage progression, overall survival (OS), cancer-free survival (CFS), and dialysis-free survival (DFS).

**Results:**

Forty-one patients underwent RN and 49 PN. RN cases had significantly larger tumors and higher RENAL complexity scores (P < 0.001). At a mean follow-up of 45.7 months, PN demonstrated a smaller eGFR decline (−13.2 ± 3.5 mL/min/1.73 m^2^) compared with RN (−23.3 ± 6.0 mL/min/1.73 m^2^) (P < 0.001), including in the subgroup with eGFR ≤ 45 mL/min/1.73 m^2^ (P = 0.002). CKD stage progression occurred in 100% of RN versus 62.2% of PN patients. Long-term OS, CFS, and DFS were comparable between groups (all P > 0.05). Age (hazard ratio (HR) 1.1) and positive proteinuria (HR 5.4) were independent predictors of overall mortality.

**Conclusions:**

PN is the preferred strategy for RCC patients with compromised renal function, when technically feasible, due to its superior functional outcomes. We propose a proteinuria-driven risk stratification approach; the presence of pre-operative proteinuria should strongly favor nephron-sparing surgery and necessitate rigorous long-term nephrological co-management to optimize survival.

## Introduction

Renal cell carcinoma (RCC) accounts for 2% to 3% of all adult cancers [1]. Due to the advancement of imaging, up to 60% of RCC cases are now detected incidentally at localized stages [2]. The standard treatment for localized RCC has shifted towards nephron-sparing surgery (NSS), primarily partial nephrectomy (PN), due to its proven benefit in preserving long-term renal function compared to radical nephrectomy (RN) [3, 4].

The management dilemma is amplified in patients with pre-existing renal insufficiency, defined as chronic kidney disease (CKD) stage III or higher (estimated glomerular filtration rate (eGFR) < 60 mL/min/1.73 m^2^), or those with a solitary kidney [5, 6]. In this high-risk population, minimizing further kidney function loss is paramount to prevent progression to end-stage renal disease (ESRD) and dependency on dialysis [7], which is associated with significant physical, psychological, and financial burden [8].

While PN is generally favored to maximize functional preservation, the long-term functional and survival advantage of PN over RN, particularly in patients with severe underlying CKD (eGFR < 45 mL/min/1.73 m^2^), remains debated in the international literature [6, 7, 9]. Some studies suggest that the functional protective effect of PN may diminish over extended follow-up in patients with severe CKD [5].

In developing nations like Vietnam, the progression to ESRD imposes a disproportionate socio-economic burden. The escalating prevalence of CKD has strained the national healthcare infrastructure, where access to renal replacement therapies remains resource-intensive. Hemodialysis, the primary modality for ESRD management in Vietnam, not only demands significant direct medical expenditures but also severely impairs the patient’s quality of life and productivity. For patients with RCC who already present with compromised renal function or a solitary kidney, the risk of post-operative dialysis is a critical concern. In this context, surgical strategies that maximize nephron preservation are not merely a clinical preference but a vital necessity to mitigate the long-term economic strain on both the healthcare system and the patients’ families.

In Vietnam, surgical resection remains the primary therapeutic approach, with PN being increasingly performed via minimally invasive techniques like conventional and robot-assisted laparoscopy. However, long-term evidence focusing specifically on the functional stability and survival outcomes of PN versus RN in local patients with CKD or solitary kidney is limited.

This study aims to comprehensively describe the clinical characteristics and identify the long-term outcomes of surgical treatment for RCC in patients with reduced eGFR or a solitary kidney in a major national referral center in Vietnam.

## Materials and Methods

This was a retrospective cohort study. The study was conducted at the Urology Department of Cho Ray Hospital, a national tertiary referral and teaching hospital in Ho Chi Minh City, Vietnam. The study period covered all eligible patients admitted from January 1, 2019, to January 31, 2024. This study was approved by the Institutional Review Board of the University of Medicine and Pharmacy at Ho Chi Minh City (IRB-VN01002/IRB00010293/FWA00023448) with approval No. 1005/DHYD-HDDD dated February 26, 2025. This study was conducted in compliance with the ethical standards of the responsible institutional committee on human subjects and with the 1964 Helsinki Declaration and its later amendments or comparable ethical standards.

The study population included all inpatients who underwent surgical resection for histologically confirmed RCC and met at least one of the following criteria: 1) pre-operative eGFR < 60 mL/min/1.73 m^2^; 2) solitary kidney (congenital, prior nephrectomy, or functional, defined as contralateral kidney function < 15% of total function on renal scintigraphy).

Patients with horseshoe kidney, or those who were kidney donors or transplant recipients, were excluded. A standardized data collection form was used to extract information on demographics, comorbidities (e.g., diabetes mellitus, cardiovascular disease), clinical presentation (e.g., flank pain, incidental detection), pre-operative laboratory data (hemoglobin (Hb), creatinine, eGFR, proteinuria), imaging findings (tumor size, RENAL score), surgical details (procedure type, technique), complications (Clavien-Dindo), and long-term outcomes (eGFR stability, CKD progression, survival).

### Definitions

eGFR was calculated using the CKD-EPI 2009 formula.

CKD stage progression was defined as an increase of at least one stage based on the KDIGO 2012 classification.

Acute kidney injury (AKI) was classified post-operatively according to KDIGO 2012 classification.

Positive proteinuria was defined as ≥ 30 mg/dL or positive dipstick test recorded in the pre-operative evaluation.

Warm ischemia time (WIT) was the time from renal artery clamping/ligation until clamp release.

Severe complication was defined as a Clavien-Dindo score of grade IIIa or higher.

Positive surgical margin (PSM) referred to tumor cells present at the cut margin confirmed by pathology.

Surgical outcome classification was based on predefined criteria: 1) good outcome: negative surgical margin, no intraoperative complications, and no severe post-operative complications (Clavien I or II); 2) average outcome: negative margin, with intraoperative complications or non-life-threatening severe post-operative complications (Clavien III); 3) poor outcome: PSM, tumor rupture recorded intraoperatively, or life-threatening complication (Clavien IV or V).

In our survival analysis, dialysis-free survival (DFS) was modeled using the Kaplan–Meier method, where mortality was treated as a censoring event rather than a competing risk.

### Statistical analysis

Data analysis was performed using SPSS version 26.0. Continuous variables were reported as mean standard deviation (SD) or median and interquartile range (IQR). Categorical variables were reported as frequencies (n) and percentages (%).

Bivariate comparisons were performed using the independent-samples *t*-test or Mann–Whitney U test for continuous variables, and Chi-squared (χ^2^) or Fisher’s exact test for categorical variables. The Wilcoxon test was used for paired comparisons (e.g., pre-operative vs. follow-up eGFR).

Survival outcomes (overall survival (OS), cancer-free survival (CFS), DFS) were calculated using the Kaplan–Meier method and compared using the Mantel–Cox (Log-rank) test. Predictors of overall mortality were identified using Cox proportional hazards regression. Correlation between risk factors and long-term eGFR decline utilized Pearson and Kendall’s Tau coefficients. A two-sided *α* of 0.05 was used for statistical significance.

Of the 90 patients initially enrolled, 73 patients (81.1%) with complete follow-up data (minimum 6 months) were included in the survival analysis (Kaplan–Meier and Cox regression). The remaining 17 patients were excluded from the long-term survival cohort due to loss to follow-up or insufficient documentation of oncological status after discharge. For the functional preservation analysis (eGFR decline), 68 patients were included, as five additional patients lacked standardized serum creatinine measurements at the 12-month post-operative milestone. Vital status was confirmed via telephone contact where possible.

## Results

**Figure 1:**
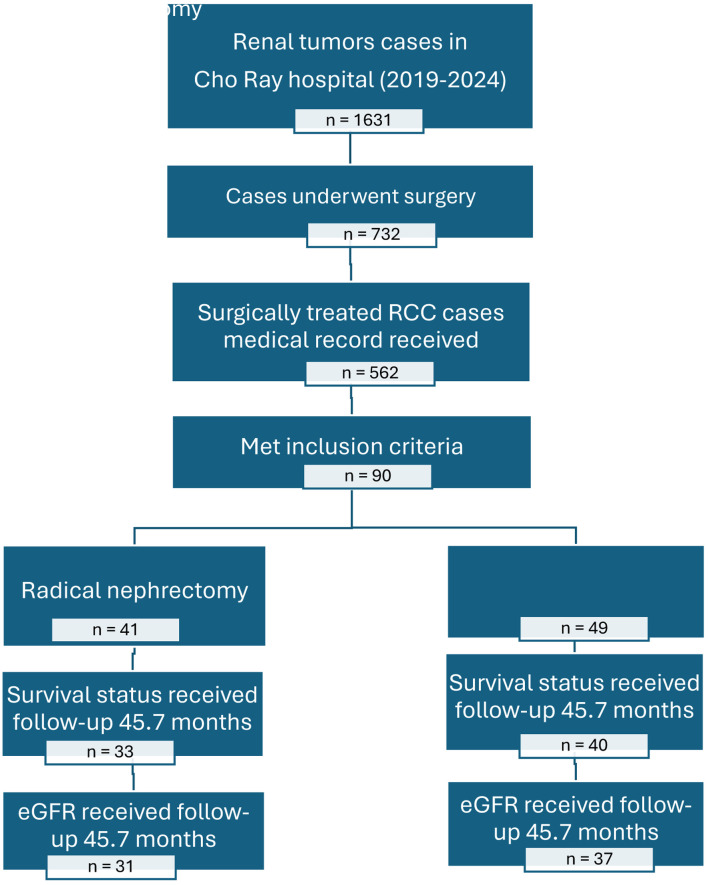
Patient selection and follow-up flow diagram for renal cancer study at Cho Ray Hospital (2019–2024). Note: Total patients (N = 90): RN group (n = 41); PN group (n = 49). Excluded from survival analysis (n = 17): loss to follow-up (n = 12); incomplete oncological records (n = 5). Final survival analysis cohort (N = 73): RN group (n = 33); PN group (n = 40). Final functional analysis cohort (N = 68): excluded (n = 5) due to missing 12-month creatinine data.

During the 5-year study period (January 1, 2019, to January 31, 2024), 90 eligible patients were included in the final analysis: 41 (45.56%) underwent RN and 49 (54.44%) underwent PN as shown in Figure 1.

The mean follow-up duration was 45.7 ± 15.8 months.

### Patient characteristics

The mean age was 61.5 ± 12.1 years. In the RN group, 78% were male with a mean body mass index (BMI) of 21.2 kg/m^2^, while the PN group consisted of 77.6% males with a mean BMI of 20.8 kg/m^2^. Flank pain (53.3%) was the most common symptom, but 41.1% of tumors were detected incidentally.

Seventy percent of patients had underlying medical conditions, most commonly cardiovascular disease (45.1%) and type II diabetes mellitus (18.9%). All four cases (4.4%) of pre-operative ESRD on dialysis belonged to the RN group.

The mean pre-operative eGFR was 46.3 ± 13.9 mL/min/1.73 m^2^, and most patients had CKD stage IIIa (68.9%). In the RN group, 19.5% patients had eGFR < 45 mL/min/1.73 m^2^, compared to 20.4% in the PN group.

Proteinuria was positive in 40% of the cohort. The RN group had a significantly lower mean pre-operative hemoglobin (121.6 ± 25.2 g/L) compared to the PN group (137.6 ± 17.6 g/L) (P < 0.001).

The mean tumor size was 5.9 ± 3.1 cm. RN tumors were significantly larger (median 8.0 cm) and more complex (mean RENAL score 10.2) compared to PN tumors (median 3.7 cm, RENAL score 7.1) (P < 0.001 for both). RN tumors were frequently stage T2 or higher (75.6% post-operative), while PN tumors were predominantly stage T1 (93.8% post-operative) (P = 0.001). Histology was mostly clear cell RCC (72.3%).

Table 1 summarizes patient demographics, clinical status, and tumor characteristics, demonstrating the selection bias in surgical indication.

**Table 1:** Baseline Characteristics and Tumor Complexity by Surgical Group (N = 90)

Characteristic	RN group (N = 41)	PN group (N = 49)	Total cohort (N = 90)	P-value
Patient demographics and pre-operative health				
Mean age (years)	59.5 ± 13.3	63.1 ± 10.9	61.5 ± 12.1	0.16
Male sex (%)	78	77.6	77.8	0.96
Mean BMI	21.2 ± 3.2	20.8 ± 2.8	21.0 ± 3.0	0.54
Flank pain (%)	-	-	53.3	-
Incidentally detected (%)	-	-	41.1	-
Cardiovascular disease (%)	-	-	45.1	-
Type II diabetes mellitus (%)	-	-	18.9	-
ESRD on dialysis (%)	9.8	0	4.4	0.042
Mean pre-operative Hb (g/L)	121.6 ± 25.2	137.6 ± 17.6	130.3 ± 22.8	< 0.001
Mean pre-operative eGFR (mL/min/1.73 m^2^)	43.2 ± 16.7	48.8 ± 10.4	46.3 ± 13.9	0.58
Baseline eGFR < 45 (mL/min/1.73 m^2^)	8 (19.5%)	10 (20.4%)	18 (20.0%)	0.92
Positive proteinuria (%)	41.5	38.8	40	0.482
Tumor and pathological characteristics				
Median tumor size (cm)	8.0	3.7	5.9	< 0.001
Mean RENAL score	10.2	7.1	8.5	< 0.001
Post-operative ≥ T2 (%)	75.6	6.2	37.7	0.001
Histology: clear cell RCC (%)	75.5	69.4	72.3	0.4

This table summarizes patient demographics, clinical status, and tumor characteristics, demonstrating the selection bias in surgical indication. BMI: body mass index; eGFR: estimated glomerular filtration rate; ESRD: end-stage renal disease; Hb: hemoglobin; PN: partial nephrectomy; RCC: renal cell carcinoma; RN: radical nephrectomy.

### Intraoperative and short-term outcomes

#### Surgical technique

RN was mainly performed via open surgery (75.6%), while PN utilized 57.1% minimally invasive techniques (PN/robot) (P = 0.001).

#### Morbidity

Mean blood loss was higher in the RN group (219.3 ± 307.7 vs. 118.2 ± 95.5 mL; P = 0.02). All five cases of transfusion (12.2%) occurred in the RN group (P = 0.017).

#### Acute functional change

eGFR decline immediately post-operatively was similar between groups (RN: −6.2 ± 9.0 mL/min/1.73 m^2^ vs. PN: −6.3 ± 7.5 mL/min/1.73 m^2^; P = 0.652).

#### AKI

AKI occurred in 31.1% of the cohort. Three cases (3.3%) required emergency dialysis, and all were in the RN group (7.3% of RN patients). The overall severe complication rate (Clavien Dindo ≥ IIIa) was 5.5%.

Table 2 summarizes surgical morbidity, technique utilized, and the initial functional response to surgery.

**Table 2:** Perioperative Outcomes and Early Functional Change (N = 90)

Outcome	RN group (N = 41)	PN group (N = 49)	Total cohort (N = 90)	P-value
Surgical technique and morbidity				
Open surgery (%)	75.6	42.9	57.8	0.001
Minimally invasive/robot (%)	24.4	57.1	42.2	0.001
Mean blood loss (mL)	219.3 ± 307.7	118.2 ± 95.5	164.2 ± 223.6	0.02
Transfusion required (%)	12.2	0	5.6	0.017
Overall AKI incidence (%)	31.7	30.6	31.1	0.286
Emergency dialysis required (%)	7.3	0	3.3	0.091
Severe complication (≥ Clavien IIIa) (%)	9.76	2.04	5.5	0.229
Functional change				
Change in eGFR post-operative (mL/min/1.73 m^2^)	-6.2 ± 9.0	−6.3 ± 7.5	−6.2 ± 8.2	0.652
Pre-operative eGFR vs. post-operative eGFR	P < 0.001 (Paired)	P < 0.001 (paired)	P < 0.001 (paired)	-

This table summarizes surgical morbidity, technique utilized, and the initial functional response to surgery. AKI: acute kidney injury; eGFR: estimated glomerular filtration rate; PN: partial nephrectomy; RN: radical nephrectomy.

### Long-term renal functional outcomes

At the study endpoint (mean follow-up 45.7 months), data were analyzed for 68 patients (31 RN, 37 PN). The overall mean eGFR decline was −17.8 ± 7.0 mL/min/1.73 m^2^. The long-term eGFR decline was significantly less severe in the PN group (change: −13.2 ± 3.5 mL/min/1.73 m^2^) compared to the RN group (change: −23.3 ± 6.0 mL/min/1.73 m^2^) (P < 0.001). This functional advantage was preserved in the subgroup with extremely low pre-operative eGFR (≤ 45 mL/min/1.73 m^2^), where RN decline was −19.3 ± 6.5 mL/min/1.73 m^2^ versus PN decline −12.4 ± 2.5 mL/min/1.73 m^2^ (P = 0.002).

#### CKD progression

Over the follow-up period, 100% of RN patients experienced CKD stage progression, compared to 62.2% of PN patients (P = 0.004).

#### Predictors of eGFR decline

Factors correlating negatively (increasing decline) with long-term eGFR preservation were: RENAL score (P < 0.001), tumor size (P < 0.001), and intraoperative blood loss (P = 0.012).

Table 3 demonstrates the long-term functional advantage of PN over RN, particularly regarding the magnitude of eGFR decline.

**Table 3:** Long-Term Renal Functional Outcomes (N = 68)

Outcome	RN group (N = 31)	PN group (N = 37)	P-value
Mean follow-up duration	45.7 ± 15.8 months		
Pre-operative eGFR (baseline) (mL/min/1.73 m^2^)	49.8 ± 6.7	48.8 ± 11.1	0.522
Long-term eGFR (endpoint) (mL/min/1.73 m^2^)	26.5 ± 7.3	36.8 ± 10.8	-
Total eGFR decline (mL/min/1.73 m^2^)	−23.3 ± 6.0	−13.2 ± 3.5	< 0.001
CKD stage progression rate in F/U (%)	100% (31/31)	62.2% (23/37)	0.004
Subgroup: pre-operative eGFR ≥ 45 mL/min/1.73 m^2^	RN group (N = 23)	PN group (N = 27)	P-value
Pre-operative eGFR (baseline) (mL/min/1.73 m^2^)	53 ± 3.9	54.5 ± 4.1	0.124
Long-term eGFR (endpoint) (mL/min/1.73 m^2^)	28.3 ± 7.2	41.0 ± 6.9	-
eGFR decline (mL/min/1.73 m^2^)	−24.7 ± 5.3	−13.5 ± 3.8	< 0.001
Subgroup: pre-operative eGFR < 45 mL/min/1.73 m^2^	RN group (N = 8)	PN group (N = 10)	P-value
Pre-operative eGFR (baseline) (mL/min/1.73 m^2^)	40.5 ± 3.5	33.2 ± 8.7	0.083
Long-term eGFR (endpoint) (mL/min/1.73 m^2^)	21.3 ± 5.1	20.8 ± 8.3	
eGFR decline (mL/min/1.73 m^2^)	−19.3 ± 6.5	−12.4 ± 2.5	0.002

This table demonstrates the long-term functional advantage of PN over RN, particularly regarding the magnitude of eGFR decline. eGFR: estimated glomerular filtration rate; PN: partial nephrectomy; RN: radical nephrectomy.

### Long-term survival outcomes

**Figure 2:**
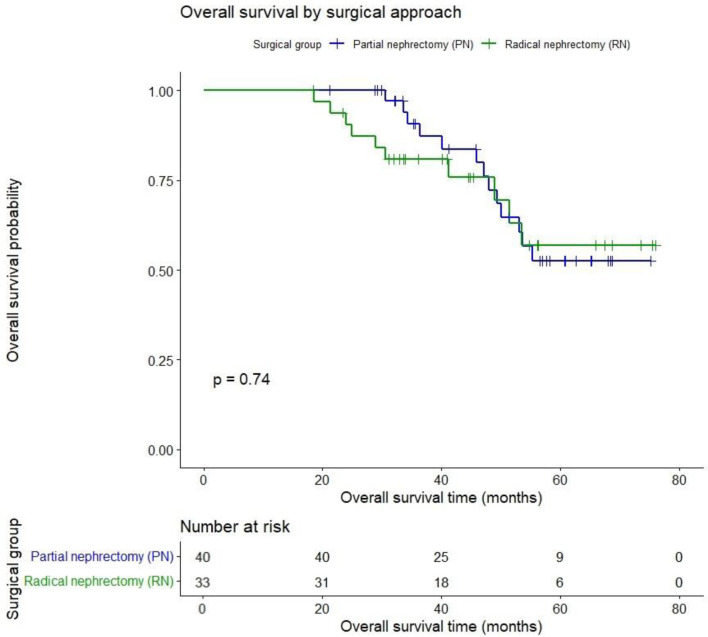
Overall survival after partial vs. radical nephrectomy for renal cancer.

Survival outcomes (N = 73) were comparable between groups. At a mean follow-up of 45.7 months, Kaplan–Meier estimated OS was PN 75% vs. RN 69.7% (P = 0.74) as shown in Figure 2.

**Figure 3:**
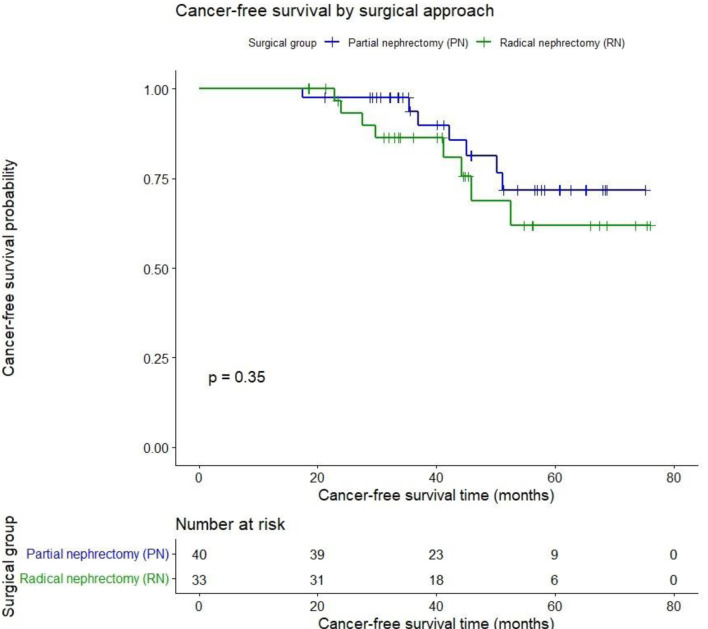
Cancer-free survival after partial vs. radical nephrectomy for renal cancer.

Cancer-free survival (CFS) after 45.7 months was PN 82.5% vs. RN 75.8% (P = 0.35) as shown in Figure 3.

**Figure 4:**
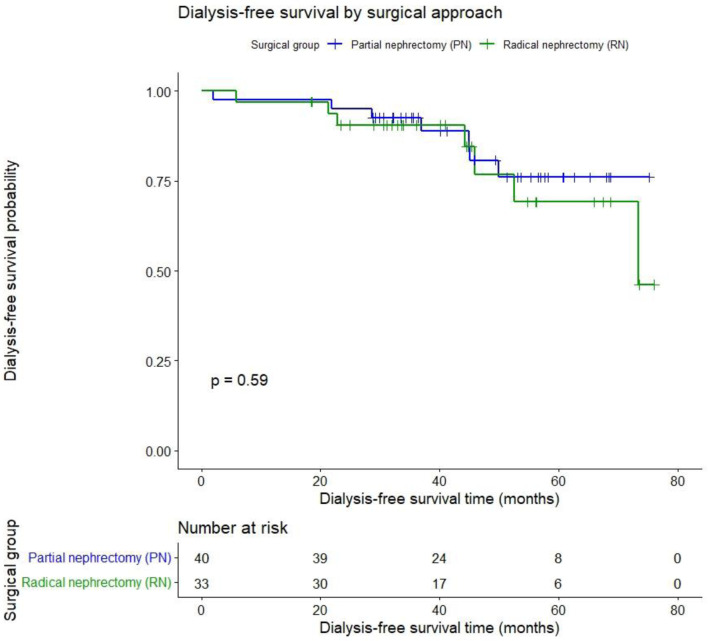
Dialysis-free survival after partial vs. radical nephrectomy for renal cancer.

DFS after 45.7 months was PN 85% vs. RN 78.8% (P = 0.59) as shown in Figure 4.

Cox regression identified two independent risk factors for mortality: age (hazard ratio (HR) 1.1; 95% confidence interval (CI): 1.02–1.12; P = 0.01) and positive proteinuria (HR 5.4; 95% CI: 1.35–21.81; P = 0.017). Pre-operative eGFR, tumor size, and RENAL score are not independent risk factors with P > 0.05.

Table 4 confirms comparable survival rates between the two surgical groups, while identifying independent systemic risk factors for overall mortality.

**Table 4:** Long-Term Survival Outcomes and Predictors of Mortality (N = 73)

Outcome	PN group (N = 40)	RN group (N = 33)	P-value
Overall survival (OS) rate (%)	75.0	69.7	0.74
Cancer-free survival (CFS) rate (%)	82.5	75.8	0.35
Dialysis-free survival (DFS) rate (%)	85.0	78.8	0.59
Independent risk factors for overall mortality (Cox regression)	Hazard ratio (HR)	95% Confidence interval (CI)	P-value
Age (per year increase)	1.1	1.02–1.12	0.01
Positive proteinuria	5.4	1.35–21.81	0.017
Preoperative eGFR	0.97	0.93–1.0	0.064
Tumor size	1.07	0.87–1.32	0.503
RENAL score	1.02	0.78–1.33	0.89

This table confirms comparable survival rates between the two surgical groups, while identifying independent systemic risk factors for overall mortality. eGFR: estimated glomerular filtration rate; PN: partial nephrectomy; RN: radical nephrectomy.

The median follow-up duration for the study cohort was 45.7 months. During the follow-up period, 17 patients (18.9%) were lost to follow-up, primarily due to geographic barriers or choosing to continue monitoring at local provincial hospitals. A comparison of baseline characteristics between the follow-up group (n = 73) and the lost-to-follow-up group (n = 17) showed no statistically significant differences, suggesting that the loss was random and unlikely to introduce significant selection bias into the survival outcomes.

## Discussion

This study presents a robust evaluation of functional and survival outcomes following surgical management of RCC in a challenging cohort characterized by low baseline eGFR, confirming the long-term superiority of PN for renal function preservation, while maintaining equivalent oncological safety.

### Demographics and clinical presentation

The demographic profile, with a mean age of 61.5 years and a strong male predominance (77.8%), aligns generally with global RCC epidemiology [5, 10, 11]. However, the mean BMI (21.0 kg/m^2^) was notably lower than in Western cohorts, reflecting regional anthropometric characteristics [6, 9]. The high rate of pre-existing comorbidities, particularly cardiovascular disease (45.1%) and diabetes (18.9%), underlines why all patients met the CKD inclusion criteria. The association between metabolic diseases and CKD is well-established [12–14].

### Surgical selection bias and tumor complexity

A clear selection bias was observed: RN was reserved for significantly larger tumors (median 8.0 vs. 3.7 cm) and tumors with higher anatomical complexity (RENAL score 10.2 vs. 7.1). The RN group comprised mostly advanced tumors (75.6% stage T2 or higher), whereas the PN group was predominantly stage T1 (93.8%). This surgical strategy is consistent with guidelines prioritizing PN for localized, less complex lesions to safeguard function, while accepting RN for advanced tumors where PN might compromise oncological control [5, 15, 16]. This strategy is further supported by the RN group having lower pre-operative Hb and higher blood loss/transfusion rates, indicative of more complex, higher-stage procedures.

### Functional preservation: the long-term benefit of PN

We acknowledge a loss to follow-up rate of 18.9% in our cohort. While this reflects the real-world challenges of long-term monitoring in a major tertiary referral center in Vietnam—where many patients reside in distant provinces—the baseline similarities between the followed and lost-to-follow-up groups help maintain the validity of our comparative findings between PN and RN.

The most critical finding is the long-term functional stability afforded by PN. While both procedures resulted in a significant drop from baseline eGFR (P < 0.001), the magnitude of eGFR decline in the RN group was twice that of the PN group (PN: –13.2 mL/min/1.73 m^2^ vs. RN: –23.3 mL/min/1.73 m^2^). This severe decline resulted in 100% of RN patients experiencing CKD stage progression.

Importantly, the benefit of PN persisted even in the highly vulnerable subgroup with extremely low pre-operative eGFR (≤ 45 mL/min/1.73 m^2^) (P = 0.002). This finding challenges some earlier reports suggesting that the functional advantage of PN might disappear in severely impaired kidneys [5, 9]. In our setting, where baseline function is low, the protection offered by every preserved nephron is maximized.

### Predictors of functional decline

Factors associated with greater long-term eGFR decline included tumor complexity (RENAL score), tumor size, and intraoperative blood loss. This reinforces the notion that meticulous surgical technique and careful selection for PN, minimizing WIT and blood loss, are crucial for achieving functional benefit. Conversely, higher baseline eGFR and contralateral kidney function were protective factors, correlating with greater eGFR preservation.

Table 5 shows the factors correlating with a greater magnitude of eGFR decline over the long term (Note: Inverse correlation coefficient means a factor is associated with a larger eGFR decline).

**Table 5:** Correlation of Factors With Magnitude of Long-Term eGFR Decline (N = 68)

Factor	Correlation coefficient (Pearson/Tau)	P-value	Interpretation (direction of decline)
Total RENAL score	−0.454 (inverse)	< 0.001	Correlates with greater decline
Tumor size	−0.500 (inverse)	< 0.001	Correlates with greater decline
Intraoperative blood loss	−0.353 (inverse)	0.012	Correlates with greater decline
Pre-operative eGFR	0.546 (positive)	< 0.001	Correlates with less decline (protective)
eGFR of contralateral kidney	0.349 (positive)	0.014	Correlates with less decline (protective)

eGFR: estimated glomerular filtration rate.

### Survival outcomes and prognostic factors

Our analysis revealed no significant differences in long-term OS, CFS, and DFS between the PN and RN groups. These findings suggest that reserving RN for larger and more anatomically complex tumors can achieve oncological parity with PN for smaller lesions. However, the comparable survival outcomes observed in the PN group should be interpreted with caution, as they are likely influenced by a lower tumor burden and a predominance of T1 disease. To further validate these trends and mitigate inherent selection bias, future multi-center studies utilizing propensity score matching are warranted.

However, the analysis identified age (HR 1.1) and positive proteinuria (HR 5.4) as strong, independent predictors of overall mortality. The high HR associated with proteinuria confirms its role as a critical indicator of underlying systemic disease burden and renal damage, significantly impacting long-term survival in this high-risk population [17, 18]. It should be noted that proteinuria in this study was assessed at a single pre-operative time point, which may have amplified its prognostic signal. We propose that a proteinuria-driven risk stratification should be integrated into the clinical decision-making process for RCC patients with pre-existing CKD. Specifically, the presence of proteinuria may identify a subgroup of patients with heightened systemic vascular and cardiovascular vulnerability, thereby mandating a stronger bias toward nephron-sparing surgery to prevent further “nephron loss” and necessitating more intensive post-operative nephrological co-management.

The overall complication rate was 10%, with severe complications (≥ Clavien IIIa) at 5.5%. The incidence of AKI was similar between groups, but all three cases requiring emergency dialysis belonged to the RN group (7.3% of RN patients). This highlights the immediate, severe functional consequence of removing the entire kidney volume in a patient with reduced reserve. PN was associated with minimal severe complications, reinforcing its safety profile even for moderately complex tumors in high-risk patients.

The high cardiovascular risk profile inherent in the CKD population further underscores the need for a comprehensive cardio-oncological evaluation before major surgery like nephrectomy. In our study, the mortality risk was significantly linked to factors like age and proteinuria, which are also established cardiovascular risk markers. Therefore, pre-operative risk stratification using advanced imaging may be beneficial. According to a recent comprehensive meta-analysis of 104 studies [19], while exercise stress testing remains a basic tool, other modalities such as stress echocardiography and cardiac magnetic resonance offer superior diagnostic accuracy for obstructive coronary artery disease. Specifically, cardiac magnetic resonance demonstrated the highest sensitivity (91%) and specificity (88%) in detecting significant coronary stenosis [19]. Integrating these high-accuracy diagnostic tools into the pre-operative workflow for RCC patients with pre-existing CKD could potentially mitigate peri-operative risks and improve long-term survival. While beyond the primary scope of this study, integrating advanced cardiac stress imaging into pre-operative risk stratification may further improve outcomes in this vulnerable population.

This study is not without limitations. Primarily, its retrospective and single-center nature introduces potential for selection bias and confounding, which cannot be entirely mitigated. A prominent surgical selection bias was evident, where RN was reserved for significantly larger and more complex tumors (higher RENAL scores), while PN was performed for smaller, less complex masses. This inherent difference must be considered when interpreting the comparable long-term survival rates.

Furthermore, while the sample size was sufficient for assessing functional outcomes, it limits the statistical power for detecting subtle differences in OS and for robust subgroup analyses among the extremely low eGFR cohort. Crucially, a key technical factor affecting post-PN function, WIT, was not uniformly recorded or available for inclusion in our multivariate analysis, precluding a more granular assessment of technical determinants of functional decline. Despite these limitations, our findings provide significant, long-term functional and oncological data on a high-risk cohort from the Vietnamese population.

A notable limitation in our granular assessment of renal functional preservation is the lack of standardized data on WIT across the cohort. WIT is a well-established intraoperative predictor of post-operative AKI and long-term functional decline in PN. Without these data, we could not fully adjust our multivariate models for the specific impact of ischemia duration on the superior functional outcomes observed in the PN group. However, it is worth noting that in our institutional practice, selective clamping or off-clamp techniques are consistently prioritized whenever technically feasible to minimize ischemic insult to the remaining parenchyma, potentially mitigating the functional impact of ischemia even in the absence of precise duration records. Consequently, future prospective studies should prioritize the precise recording of WIT and the use of specialized techniques, such as zero-ischemia, to further delineate the drivers of functional recovery in CKD patients. Furthermore, applying competing-risk models in subsequent research would provide a more nuanced assessment of dialysis dependency in the presence of mortality.

In conclusion, our study demonstrates that PN provides superior long-term renal functional preservation compared to RN in patients with pre-existing CKD, without compromising survival outcomes, when technically feasible. We identified age and positive proteinuria as independent predictors of overall mortality in this high-risk cohort. These findings support the implementation of a proteinuria-driven risk stratification strategy, where the presence of pre-operative proteinuria should strongly favor nephron-sparing approaches and necessitate rigorous post-operative nephrological surveillance. Such a personalized clinical approach is essential to optimize both oncological and systemic health outcomes in this vulnerable patient population.

## Data Availability

The data supporting the findings of this study are available from the corresponding author upon reasonable request.

## Abbreviations

AKI: acute kidney injury
BMI: body mass index
CFS: cancer-free survival
CKD: chronic kidney disease
DFS: dialysis-free survival
eGFR: estimated glomerular filtration rate
ESRD: end-stage renal disease
OS: overall survival
PN: partial nephrectomy
PSM: positive surgical margin
RCC: renal cell carcinoma
RN: radical nephrectomy
WIT: warm ischemia time
